# Extracts and Abstracts

**Published:** 1889-02

**Authors:** 


### EXTRACTS AND ABSTRACTS.

***Medical Treatment of Fibroid Tumors of the Uterus.***—Dr. Bedford Brown, of Alexandria, Va., read a paper on this subject before the Southern Surgical and Gynæcological Association in December, 1888. In regard to treatment he says: In the process of tumor building there are manifestly two opposite pathological conditions, one a positive, the other a negative character, existing. These conditions consist of increased cell proliferation and organic growth, and deficiency of tissue metamorphosis to counterbalance the act of superabundant deposition. Therefore, whatever in the way of diet, regimen, exercise, period of life or other circumstances may tend to increase cell proliferation beyond the powers of nature to maintain an equilibrium, will, when brought to bear upon the uterine structure, promote the fibroid growth. On the contrary, whatever of food, habits, medicine or period of life will correct this perverted state of the nutritive processes of the part will diminish abnormal cell proliferation and restore healthy tissue metamorphosis, and must curtail fibroid growth.

I think there can be no question that discrimination in regard to the character of the food used by these subjects exerts an influence over the growth of fibrous tumors. This accords with my own experience. The curtailment in supplies of those varieties of food containing largely, if not wholly, of amylaceous and fatty materials does retard these growths. On the contrary, they grow more rapidly when starchy and fatty matters are more largely consumed. Lean fresh meats, green vegetables and fruits containing but little starch, skimmed milk and eggs, I have found effected this object best.

Then the power of ergot to diminish the calibre of the nutrient vessels of the uterus, and in this way curtail the hypernutrition and growth of the tumor is so well understood as to require scarcely a notice here, barely excepting the special preparations of that article and the method of administration. I have found the so-called normal liquid ergot of Parke, Davis & Co. an excellent preparation for internal or hypodermic use, and I regard it as particularly well adapted to the latter. It is very unirritating, and acts with great promptitude in arresting hæmorrhage even of the greatest magnitude. I believe that the continuous internal use of this article in twenty or thirty drop doses three times daily will accomplish more in the end than the hypodermic method. With this may be alternated a pill after the following formula:

℞.

Ergotini.................ʒij

Strychninæ...............gr. j

Quininæ salicylat..........ʒiss

Acidi arseniosi............gr. j

1 gr. pil xxx.

One of these should be taken three times a day in connection with the following combination of remedies:

℞.
Flu. ext. hydrastis can.......℥ss
Aquæ cinnam..............℥v
Flu. ext. phytolaccæ, decand.℥ss
Sodæ bicarb...............ʒij
M.

These remedies in this particular combination I have found useful in retarding and diminishing the growth of fibroid tumors.

The hydrastis and phytolacca both possess properties which give them power to retard abnormal uterine action and promote absorption. But I am persuaded that there are other therapeutic agents which, acting on the blood formation and through the circulation, influence the process of nutrition, rectifying that important function, when deranged, in a remarkable manner, and in that way correcting those excesses of local action which produce local disease and abnormal growths. Some years since, with a view of carrying into practice this idea, I began a series of experiments for the purpose of testing the influence on the progress and development of fibrous tumors by acting on the nutritive functions by means of those preparations containing phosphorus, lime and soda. I had at that time four cases of fibroid under my care. I selected to be used in these cases the syrup of the lacto-phosphate of lime and the syrup of the hypophosphites of lime and soda, as prepared by McArthur. That was about eight or nine years ago. Since that time I have had nine cases under my care. Six of these cases have remained under my inspection, and with one exception are so decidedly improved as to need no further treatment. The one exception came under my care during the spring, and is now improving under treatment. Three of these cases alluded to have left my vicinity and migrated to a distance. In these cases the syrup of the lacto-phosphate of lime and the syrup of the hypophosphites were administered in teaspoonful doses each three times a day continuously for months, and with slight intervals, so as to give rest to the stomach, for years, that there might be maintained on the system a permanent influence.

The character of most of the cases subjected to this treatment was of the most aggravated form, and had previously been treated by means of ergot internally and hypodermically, the bromides, etc., without in any way retarding their progress. In some constitutions the response to treatment is much more prompt than in others. Some cases improve up to a certain point, then cease and wait for the menopause to complete the process. Some recover entirely before that period, while others again are very resistant to treatment in any form. But the treatment by means of the phosphates has given me far more satisfaction than any other.

This treatment to produce favorable results must be pursued systematically, regularly, perseveringly and continuously. Taking a retrograde review of the cases treated by myself, I will state here that the first two cases which came under my charge about thirty-five years ago, in absence of knowledge of better remedies were both treated by means of liquor arsenici et hydrargyri iodidi in one and two drop doses continued for two or three years with occasional intermissions with a view to its alterative effect. The tumor in both of these cases was in the cavity of the uterus and submucous in character. The females belonged to the negro race and had borne children. In both there existed decided menorrhagia and one was subject to periodical hæmorrhages. In both cases also the uterus was distended so as to fill the cavity of the pelvis.

In the worst case after three or four years' treatment by this remedy the tumor began to diminish and finally became entirely absorbed. The other improved but did not recover entirely until after the menopause. Ergot was also used occasionally in both to repress hæmorrhages. It was about this period that Dr. Simpson introduced his treatment by means of the bromide of potash. I continued to use the treatment of Simpson I thought with some benefit, in connection with ergot up to the period when I adopted the use of the phosphates. Subsequently to these two cases I tested further the powers of the solution of Donovan, but was not so fortunate. The therapeutic action of the combination of the lacto-phosphate of lime and hypophosphites in the seven last cases coming under my care, has been exceedingly favorable and has been of a two-fold character.

The earliest perceptible effect exerted was on the tendency to hæmorrhage. In every case this tendency has been diminished and so decidedly lessened in most of them as to afford relief. In two cases, one of which was the worst in this respect that I ever witnessed, the hæmorrhages were frequent and often dangerous in extent. This patient after taking these remedies for three or four months found herself without her usual hæmorrhages, and not long after found herself minus her menstrual periods. She at once became alarmed and protested that she would take no remedy which would affect her in this way. She had a firm conviction that her condition was due to the remedy used. It was suspended and after a few months the hæmorrhages returned. The remedy was a second time recurred to, and with a similar effect, and again suspended. Under treatment by the phosphates, and occasionally ergotine, and strychnine, the hæmorrhages finally ceased and the absorption of the tumors, there were three of them, progressed.

In the case of another patient while under the phosphate treatment a suspension of menstruation occurred and this patient refused to continue their use. While the tumor in this case was large and impaired the general health seriously, there were no hæmorrhages. The second effect of the phosphates in these cases is to promote absorption and arrest the growth of the fibroids. In all these cases there was arrest of growth and diminution. In several, to be more definite, in three, the diminution was very marked. In this case there was complete absorption. Thirdly, the action of the combined phosphates in these cases, in which there is always impairment of the general health, is to improve the condition in a manifest degree of the constitution and in every case hâve proven themselves genuine restoratives. In these cases in which our greatest renovator, *iron*, is inadmissible the phosphates fill a most important place. Following is an illustrative case:

*Case I.*—Mrs. M——, a lady, aged about 32 years, the widow of a confederate officer who fell at the battle of Seven Pines only two months after marriage. She has never borne children. She had a submucous fibroid distending the uterus to the full capacity of the pelvic cavity. Her general health was much impaired from the depressing influence of hæmorrhages, pain and local irritation. I presumed that the tumor had been in progress at least four or five years. Various medicines as ergot, the bromides, and iodides with tonics were resorted to without benefit. About this time from some cause not well defined extensive bronchitis with some indications of hectic appeared. For this complication McArthur's syrup of hypophosphite was used which was subsequently united with the syrup of the lacto-phosphate of lime. The symptoms arising from the tumor improved so much as to justify their continuation. After a time all hæmorrhages ceased and menstruation became regular and the tumor began gradually to diminish and in two or three years of this treatment became so greatly lessened as to give no trouble. This patient subsequently removed from the city to a distant point. This was the first case in which I used the lacto-phosphates and hypophosphites in fibroid tumor of the uterus.

In the cases of excessive hæmorrhage it is our duty not only to adopt means to arrest the immediate attack, but to go further than that and so shape our treatment as to control or subdue that tendency. I believe the phosphatic treatment persistently pursued; the ergotine and strychnia internally, and the local application of the iodine and the extract or normal liquid ergot alternately every third day will so impress the general and local circulation and the process of nutrition as to regulate this peculiar tendency.

I attach very considerable importance to the influence of local treatment applied in this way as a means of aiding in controlling hæmorrhage, and promoting the reduction and absorption of the fibroid. The iodine and ergot are both readily absorbed and exert their peculiar influence with more or as much force than when taken internally. At all events in my hands the practical results have been good.

After witnessing the really marvelous effects of these agents in acute-glandular affections I am constrained to believe that they must exert a very great influence on the functions of that system generally as well as on the process of metamorphosis of tissue by promoting its disintegration and removal. In the administration of the hypophosphites and lacto-phosphates in cases of grave malnutrition giving rise to serious local lesions, excessive exudation of morbid products or of abnormal cell proliferation producing unwonted tissue growth endangering health and life, I have found them to accomplish more than any other remedies. I wish to dwell more forcibly and specially on the necessity of the rapid and thorough *saturation* of the circulation with the phosphates of lime and soda, as a means of restoring the lost equilibrium of nutrition, arresting increased cell proliferation and exudation and promoting their absorption. Therefore, I contend these agents have not been given in sufficient quantity heretofore to regulate the process of nutrition. I have in grave and serious cases administered as much as a drachm of the syrup of the hypophosphites every two or three hours and two drachms of the lacto-phosphate three times daily until the object was attained. In summing up the results of medical treatment of fibroid tumors, it may be stated, that the continuous administration of ergot in the form of ergotine or fluid extract, does retard the fibroid growth, does lessen the tendency to hæmorrhage, and in certain stages of fibroid growth, on occasion of hæmorrhage, given hypodermically or per arm will arrest it. But at an advanced stage, when the growth of submucous fibroid becomes very great and the walls of the uterus have become very thin and attenuated from over-distention and absorption and paralysis of uterine muscle, then the ergot ceases to act as an hæmostatic. The fluid extract of hydrastis does act on the growth of fibroid to a limited extent by diminishing the circulation and also lessens the tendency to hæmorrhage. In my experience it acts better when given in combination with the phytolacca decandra.

Local treatment by means of iodine and ergot applied extensively over the cervix and roof of the vagina give important aid in promoting the same therapeutic results.

By a judicious and timely combination of the various therapeutic agents mentioned I believe the great majority if not all cases of uterine fibroid may be shorn of their worst features; that the sufferings of those so afflicted may be greatly alleviated; that life may be prolonged; that all may be benefited and certain proportions cured. I am in a position to assert truthfully that benefit has resulted from treatment in every case when the patient was under charge sufficiently long to get fully under the influence of medical treatment. Hence, I am convinced by a long and not very inconsiderable personal experience that medicine can do much and is capable of doing still more in the future for the benefit and relief of these cases.

---

***Locking, Retroversion and Strangulation of Uterine Fibroids in the Pelvic Excavation.***—At the meeting of the Obstetrical Society of London on November 7, Dr. J. Matthews Duncan, in a paper on this subject, stated that locking in the pelvic excavation implied impaction not the result of adhesions. Its effects might be produced by pressure into the pelvic brim of a tumor too large to pass into the excavation. Retroversion of a fibroid closely resembled the retroversion of the gravid uterus in its characteristic form. The symptoms and treatment of the two conditions were nearly alike. Strangulation, with locking, of a fibroid, with or without retroversion, was a rare accident; a case was described. Dr. Duncan had not seen a similar case of strangulation of or by a gravid uterus.

Mr. Meredith described *A Case of Locked Fibroid, treated by Supra-Vaginal Hysterectomy:* A single woman, aged 36, was admitted into hospital under Dr. Percy Boulton last May. A uterine tumor was firmly impacted in the pelvic cavity. For nine months the pressure of the growth on the neck of the bladder had led to frequent attacks of complete retention. The recurrence of these attacks had latterly been avoided only by relieving the bladder at regular intervals of not less than two hours, both by night and by day. After unsuccessful attempts to dislodge the tumor from the pelvis by vaginal taxis, the case was transferred to Mr. Meredith's care, with a view to abdominal section. At the operation, performed by him on June 2nd, considerable difficulty was experienced in extracting the impacted mass, which, together with the uterine body, and its appendages, was subsequently removed by supra-vaginal hysterectomy. The tumor weighed two pounds, and was of the size of a large cocoanut, and consisted of a densely-packed mass of fibro-myomatous growths developed in the posterior wall of the uterus. The after-progress of the case was uninterrupted, and the patient made an excellent recovery, leaving the hospital exactly six weeks from the date of operation with the abdominal incision soundly healed throughout.

Dr. Gervis drew attention to the usefulness of a suitable pessary in preventing a recurrence of the downward displacement of a fibroid after it had been pushed up out of the pelvic cavity. He felt satisfied with hydrostatic pressure in cases where the taxis had failed, noting a case in his own practice resembling Mr. Meredith's.

Dr. Graily Hewitt found that upward pressure and properly adapted vaginal support often proved sufficient to relieve impaction. In some cases impaction of a large fibroid was slow to cause difficulty in micturition; in others that trouble rapidly set in when the impacted tumor was small. In a very marked case a fibroid growth at the back of the uterus occasioned sudden impaction with retroversion and enormous distention of the bladder. Dr. Hewitt thought that cases of anteversion with hypertrophy of the uterus were sometimes mistaken for fibroids. In one instance proper diagnosis and appropriate treatment cured a case of this kind after several years of suffering. In another case, where an egg-shaped tumor grew anteriorly, a little to the right side, a well-adjusted pessary was very successfully used and the tumor raised out of the brim. Its pressure had rendered the patient a complete invalid.

Dr. Lewers referred to a case where a uterus retroverted by fibroids caused retention of urine in a woman, aged 49. The urine was drawn off, and the uterus replaced bimanually; then a large ring pessary was introduced. This was done two months ago. Dr. Lewers had seen the case recently, and found the uterus in good position; the patient had no trouble with her water. He mentioned this case because he gathered from Dr. Duncan's paper that, in similar instances, replacement, even when possible, was usually followed by recurrence of the malposition.

Dr. Aust Lawrence (Clifton) found that in one case of retroflexed gravid uterus and in two of "locked fibroids" he was enabled to effect reduction by keeping the patient in bed in the semiprone posture for twenty-four hours. Without this kind of treatment, repeated attempts at replacement should never be made.

Dr. Champneys advocated hydrostatic pressure exerted by gravitation. He had found it succeed where taxis had failed, and he always used it after the failure of taxis before proceeding further. The mode of using it had been described in the *Lancet* some years back. It was conveniently employed by means of a child's air-ball connected with an irrigator. Any desired amount of even and continuous pressure could be applied and removed at will. A fibroid might be impacted for many reasons, such as bulk, œdema, adhesions, and expansion of the broad ligament. An air-tight adaptation was an obstacle to replacement; in raising a tumor in abdominal section a loud sucking sound was often heard. Hydrostatic pressure got rid of some of the œdema, adhesions could not be rudely torn, as by taxis, and the broad ligaments would be unaffected. Thus the method furnished a valuable differential prognosis as to the possibility of replacement. He did not say that the tumor was replaceable in Mr. Meredith's case, but he should himself have tried hydrostatic pressure before resorting to abdominal section.

Dr. Priestley remarked that Dr. Duncan's experience proved that, whether the pressure were made with the fingers or with hydrostatic bags as Dr. Champneys had suggested, and aided by the genu-pectoral position, difficult cases of impacted fibroid might be overcome provided that the pressure was made in the right direction for a sufficient period. Dr. Priestley thought that the alleged frequency of absolute impaction was overrated. The symptoms produced by pressure almost amounting to impaction were more frequent; these were retention of urine in certain cases, incontinence of urine in others. He had recently seen a patient who had a bulky fibroid, and was constantly losing large quantities of limpid fluid, supposed, at first, to be from the uterus, but ultimately proved to come from the bladder. Not only retroversion of a uterus bearing a fibroid could cause impaction, but also some forms of fibroid without backward displacement, particularly those ovoid forms in which the lower segment fitted closely into the brim and cavity of the pelvis. He had sometimes been surprised how small a degree of force exercised in pushing up the tumor from below would bring at least temporary relief. Prolonged and persistent efforts, with every precaution, should always be made to reduce the tumor, as Dr. Duncan advocated, before so grave a step as abdominal section was undertaken, although Mr. Meredith's case had proved so signally successful.

Mr. Meredith stated that the absolute fixation of the pelvic tumor in his case, resisting all the repeated attempts to displace it, afforded ample justification for abdominal section as the only means of relieving the patient. In his case atmospheric pressure had not much to do with the difficulties which he encountered. After partial dislodgement of the firm, incompressible tumor from the pelvic cavity, extraction was found to be impossible until some amount of enucleation had been practiced on the left side. This fact alone proved conclusively that nothing short of operation could have proved successful in affording even temporary relief to the patient's sufferings.—*British Medical Journal*, Nov. 17, 1888.

---

***Arthrectomy versus Excision of the Knee.***—At the meeting of the Harveian Society of London on November 1, Mr. Page read a paper on this subject. After reference to the reintroduction of the operation of excision of the knee by Sir Wm. Fergusson in 1850, and the success which attended it in the hands of many surgeons soon afterwards, the reader pointed out that the operation had subsequently fallen somewhat into disfavor because of the shortening which was found to result when the operation had been undertaken in early life. At the same time the propagation of the principles advocated by his text was leading surgeons to open and examine the insides of joints at a much earlier period in cases of disease, and they had thus been induced to remove diseased structures alone rather than perform the old excision. This had largely tended to make excisions of the knee much rarer than they had been, and the object of the paper was to point out the advantages and the importance of arthrectomy or excision of diseased structures at a sufficiently early period, so as to render any true excision unnecessary. An endeavor was made to point out the kind of cases suitable for operation—those which are more often seen amongst the poor than the well-to-do—but that there should be no indiscriminate arthrectomy in all cases, but only in those where rest was impracticable or had already been tried and found wanting. The use of Thomas's splints had vastly improved the non-operative treatment of joint diseases, but nevertheless there were cases where the greatest benefit would be derived from an early excision and examination of the joint, so as to remove diseased structures only, whether in synovial membranes, cartilages, ligaments, or bone. By this plan not only were the parts about the joint placed in such a position that repair was possible, but there was also this advantage, that the tissues no longer provided a suitable nidus for the tubercle bacillus, even if that organism were not the real cause of the disease or were not already present in the joint. Remarks were also directed towards the result which it was desired to obtain—ankylosis, or a movable joint—and the reader spoke strongly in favor of ankylosis as being the best thing to be attained. It seemed a bad thing to attempt to gain movement by early manipulation, and before the parts were soundly healed, a practice not hitherto rewarded with much success, and in itself not free from risk. When movement was sought to be obtained, it was feared that the cavity of the joint might not be as thoroughly inspected as it ought to be, and diseased tissues might therefore be left behind. The object of the operation was thus not attained. Cases of Sendler and of Tilling were referred to in connection with this question of ankylosis or mobility. Mobility could only be expected in cases where the amount of disease was limited in extent, the very cases where there was difficulty in diagnosis, and where there would be hesitation to operate early. It would come to this, therefore, that in the great majority of cases which came to operation ankylosis would be the thing to aim at. The removal of diseased structures alone rendered it quite unnecessary to encroach on the growing ends of the bones, and there would be no shortening of the limb afterwards. In this respect arthrectomy was clearly a great improvement on excision, an operation which would have to be reserved for cases where there was ankylosis in a false position, or where, on opening the joint, the disease was found to have passed beyond the stage in which a mere arthrectomy would suffice. This again pointed to the advisability of early operation, unless there were the most distinct evidence of improvement of rest, for it was most desirable not to have to undertake in early life an operation at all like that of the old excision. At the close of the paper several cases were shown, and it was pointed out that some flexion which had occurred in two of them was really due to the plaster-of-paris splint having been left off too soon, and how necessary it was in all cases to maintain support to the limb until ankylosis was secure.

Mr. Edmund Owen remarked that in operating it was necessary to make such an opening into the joint that every synovial crevice could be inspected and thoroughly dealt with, and for this purpose nothing in his opinion served better than the horse-shoe incision traversing the patellar ligament. In the knees on which he had operated, and which were nearly all in an advanced stage of the disease, he had taken away the crucial ligaments, and only by doing this could one reach that part of the capsule which covered the upper and back part of the condyles. He agreed with Mr. Page that in the present state of knowledge it was better not to think of securing future movement for the joint; that if the surgeon's mind were occupied with such thoughts, he was apt to deal less thoroughly with the articular cavity than he would do if his object were to secure a straight and rigid limb. Certainly to advise that movements be imparted to the joint before healing was complete, as some seemed tempted to do, looked like courting disaster. And not only should the limb be kept rigidly extended for months, or even years if necessary, but the patient should also be under prolonged and careful supervision. The cases which Mr. Page showed were indeed a promising group, and some had borne the test of time extremely well; one must not flatter one's-self that because a child went out of hospital with a straight limb after arthrectomy that the case must be written down in the notebook as "cured." Unfortunately this was not so, and his (Mr. Owen's) experience had been that in certain unhappy cases amputation had, after hesitation and regret, to be resorted to at last. The practical surgeon was anxious to know precisely which were the cases best suited for arthrectomy; children with chronic abscess and displacement at the joint, though generally deserving of trial, were those least suited for the procedure. But on the other hand, if one operated only in the early stages of the disease there was a risk of resorting to active interference where no such measure was needed. To admit that the slight cases gave the best results was not to pay the highest tribute to the operation; and if arthrectomy were too lavishly resorted to it would be expedient or even necessary to invite the attendance of Mr. H. O. Thomas at the Society to call attention to the fact that absolute and continuous rest for a joint, together with the adoption of constitutional measures, was a well-tried means of successfully dealing with chronic disease of the knee in children.

Mr. Sheild agreed with most of the opinions expressed by Mr. Page. Absolute immobility after operation was the main factor in success after either excision or arthrectomy. He had found actual cautery useful in the treatment of oozing from cavities.

Mr. Barker hoped that ultimately, by means of arthrectomy, movable joints might be obtained. However, at present it seemed advisable to keep the limb at rest for a long time. The question of movement seemed to depend upon the choice of cases; in those which now presented themselves there was so much destruction of the cartilage, that movement could not be hoped for. Contrary to the opinion of Mr. Page, suture of the patellar ligament ought to be practiced. He had found that shock, due to prolonged operations, was diminished by the use of hot water instead of the ordinary cool carbolic douche, which had also other disadvantages.

Mr. Pick had been in the habit of performing a modified arthrectomy for fifteen years. He removed a thin layer of bone and cartilage, and all the synovial membrane. His actual arthrectomies had given him trouble because of flexion. The whole cartilage and crucial ligaments had not been removed in these cases. It was important to preserve the crucial ligaments unless they were diseased. The tendency to flexion lasted for years. Mr. Page had got the best result in the case in which he had removed the cartilages. The other cases still required supports, and he wished to know how long this must be continued. —*British Medical Journal*, Nov. 17, 1888.

---

***Nerve Stretching in Leprosy.***—Dr. Beaven Rake reports 100 operations performed on 60 patients. His paper (*British Medical Journal*, December 22, 1888) forms a part of a Government report on leprosy in Trinidad, and the operations were undertaken in consequence of some reports from India, in which the value of nerve-stretching in leprosy was spoken of very highly. Lawrie reported 30 cases in which the operation was done for anæsthesia, and says the operations were all successful. Downes has reported 32 cases of leprosy in Kashmir in which the operation was done. He says that all the cases were benfitted, and sensation restored almost to its normal condition.

In Rake's cases perhaps the most striking results were obtained in those cases in which the operation was done for pain, especially pain associated with perforating ulcer. In two of the cases the pain was so severe that the patients begged for amputation; but after the nerves were stretched the pain vanished almost immediately. In another case, in which the right sciatic was stretched for painful perforating ulcer of the foot, the ulcer became cleaner, the pain vanished, and the relief was so great that the patient asked to have the operation performed on the left side for a similar condition of that foot. Here, however, the result was not so satisfactory, for, although the ulcer became a little cleaner for a few weeks, gangrene eventually set in in both feet, and the patient died five months later.

In another case there was temporary relief from pain, but the ulcers became gangrenous, and the leg was amputated at the patient's request. The posterior tibial nerve was greatly thickened, and gelatinous looking. The stump healed in two months, and the patient was soon about the ward on a wooden leg. In two cases in which the pain recurred four months and one year respectively after the sciatic had been stretched, the external popliteal of the same side was stretched with good result. In one case of supra-orbital thickening and neuralgia, probably associated with syphilis, mercury and iodide of potassium were given for four months without much result, after which the supra-orbital was stretched with immediate relief to the pain.

The operation was done for anæsthesia in 33 cases, but the operator cannot say that the results were very encouraging. In many cases no effect whatever was produced; indeed, in some old-standing cases the nerve was exposed without chloroform, and stretched, tapped, or pricked without the least complaint of pain or even of sensation. In some of the earlier cases there seemed to be some improvement of sensation a year after the operation, and in several other cases slight temporary improvement was noted. On the whole, however, the results cannot be considered satisfactory. In one or two cases improvement of sensation has been noted a little over a year after the operation.

In 18 cases nerves were stretched with the object of watching whether any trophic effect would be produced on the growth of tubercle or general infiltration of the skin. Comparative measurements of fingers on the two sides were made, but no effect whatever could be traced to the operation.

In two cases the stretching seemed to facilitate the separation of necrosed bone. There was increased discharge from the sinuses, and fragments of bone were removed ten days and eleven days respectively after the operation. After this the ulcer nearly healed in the first case, and quite in the second.

In regard to the general results of the operation, there was more or less relief in 47 cases, no relief in 49 cases, and a doubtful result in 4 cases. Roughly, then, in 100 cases operated on, without any particular selection, it may be considered that more or less relief was given in half. The nerve, when exposed, was found to be enlarged in 48, or almost half of the cases; in 34 it was not enlarged, and in 18 its condition was not noted. The most commonly enlarged was the median; after that the ulnar and external popliteal. The sciatic was never found thickened.

Primary union was not obtained in many cases after the operation. Strong carbolic acid was applied to some of the more thickened nerves, and some of them were even scarified longitudinally, with the idea of destroying the bacilli to some extent, but no better result was noted after this procedure.

In the necropsies of some patients that died from other causes at longer or shorter periods after the operation, no naked-eye changes were found in the nerves that had been stretched, nor were any adhesions found, even when the incision had granulated up from the bottom. In one case only after operation the patient complained of pain beginning in the cicatrix over the sciatic, and traveling down the thigh. Dr. Rake concludes that the sciatic is the most satisfactory nerve to stretch, for it is nearest the spinal ganglia and commands the supply of the whole leg and foot and back of the thigh. The chief indications for the operation are perforating ulcer, some cases of pain and necrosis whether associated with perforating ulcer or with peripheral neuritis without ulcer.

The most probable explanation of the influence of nerve-stretching on ulceration, neuralgia, or other conditions seems to be that given by Mr. Marshall in his Bradshawe Lecture in December, 1883, and based on the experiments of Mr. Victor Horsley. The latter has shown that when the sciatic nerve is stretched, the stretching is observed to affect the sacral plexus, and passes to the roots of the nerves, disturbing the dura mater and shaking the cord through the ligamentum denticulatum. Thus the ganglia of the cord are probably affected, and an excitation of the vasomotor centres or trophic centres is produced. Dr. Rake has repeated the experiment on the dead body, and found that when the lumbar dura mater was exposed from behind, no movement was to be seen on stretching the sciatic nerve. But when a window was cut in the dura a distinct downward movement or dragging of the cord was seen when forcible tension was exerted on the sciatic.

This theory seems to be supported by the following facts: 1. Stretching of the sciatic seems to be attended with better results than the stretching of such a nerve as the median, which is nearer the periphery. 2. Sometimes after stretching a nerve on one side of the body, sensation seems to be improved on both sides; which looks as if a greater nerve.storm had taken place, the ganglia of both sides being shaken or affected. This might of course depend on various factors, such as greater force used in the stretching, or looser sheaths of surroundings of the nerves.

***Treatment of Phthisis.***— BERLATERS reports (*Centralblatt für Klinische Medicin*, September 8, 1888) 8 cases of tuberculosis treated with *aniline.* Of these 4 were in the early stage, 3 in the beginning of the last stage, and 1 was so far advanced in the disease that his death was expected in a few weeks. All these cases were cured or improved under the aniline treatment. The fever disappeared, the appetite was better, there was increase of weight, improvement of physical signs, and there were fewer bacilli in the sputa. The aniline was given in doses up to 12 drops, with a few drops of alcohol, and by inhalation of 25 or 30 drops. Some of the patients took, altogether, as much as two and a half ounces of aniline. The administration of the remedy should be interrupted for a few days from time to time, especially if the patient complain of lassitude and weakness in the legs. A greenish-yellow color of the skin may appear, but this disappears when the aniline is withheld for a few days.

GAGER has recently published records of 17 cases of phthisis treated with *hydrofluoric acid.* For an inhalation-chamber he used a compartment of a wooden hut, well boarded, with a close-fitting window-sash and door; its capacity was about 250 cubic feet. In this space the patients, one, two, or three at a time were seated, their clothes being protected by sheets from the injurious effects of the acid. The gas was manufactured in an adjoining chamber, and conveyed to the ceiling of the inhalation-chamber by a leaden pipe.

As a rule the patients were ordered one sitting of an hour's duration daily; occasionally two sittings were given. In order to ventilate the apartment it was always necessary to open the door and window from three to eight times during the hour.

All the patients subjected to this treatment presented tubercle bacilli in their sputa; and Gager's investigation was specially directed towards the antibacillary property of the acid.

All renal cases were excluded from this treatment. During the first three sittings all the patients complained of smarting and itching in the nose, of smarting in the eyes, and often of sneezing, which lasted for days. With some patients there was increase of cough, and even streaks of blood in the sputa; and the inhalations had to be stopped for some days on that account.

Of the 17 patients, 13 found their appetite increased after the inhalations. In one case there was slight epistaxis.

In 5 cases the bacilli disappeared from the sputa, and there was a marked improvement in the symptoms. In 7 cases the physical signs were distinctly improved. In 12 cases the body weight was increased, but the amount of increase appeared to bear but little relation to the improvement in the general condition; for example, in one case in which the bacilli disappeared and in which the physical signs improved there was no increase in the weight at all, and in another there was no improvement, though the weight increased almost four pounds.

Three of the patients had pyrexia; one lost it entirely, together with all the bacilli and expectoration; in the second case the fever decreased, and in the third it continued as high as ever. One of the patients had night-sweats, but they disappeared entirely.

In 7 cases the vital capacity increased to the extent of from 3 to 27 ounces. In 2 cases somewhat severe irritation of the laryngeal mucous membrane was set up, thus showing that this method of treatment is contra-indicated in cases of laryngeal phthisis—at all events, only a very small quantity of the gas can be given.

In 5 cases, including one in which laryngeal complications existed, no improvement could be noticed; one very advanced case died. No evil consequences were presented in any of the cases.—*Lancet*, Sept. 22, 1888.

---

***Congenital Lateral Deviation of the Eyes.*** —At the meeting of the Ophthalmological Society of the United Kingdom on November 8, MR. SWANZY, of Dublin, narrated a case of conjugate lateral deviation of the eyes, probably due to a congenital lesion. The patient was a healthy child one year old. Both eyes were turned to the right, but could be turned to the left with an effort, yet not as far as the canthi, and when they passed the middle line the effort was attended with nystagmic motions. The associated action of the interni for the purpose of convergence was unimpaired. The vision was good, and the ophthalmoscopic appearances normal. After birth, the labor being natural, the child had not opened its eyes for four days, and from that time until it was two months old there was marked nystagmus in all positions of the eyes. From that age the relations, including the father, noticed the condition described, but the mother did not do so until the child was six months old, and after it had had a fall on the right side of its head, which produced a bruise. This fall was not followed by any head symptoms. Mr. Swanzy regarded the case as due to an intra-uterine lesion situated in the pons and implicating the nucleus common to the sixth and third nerves on the left side. The symptoms might have been caused by the fall producing a cortical lesion in the right cerebral hemisphere, but experience caused doubt whether a conjugate deviation due to a cortical lesion would be so permanent a symptom, while its permanence as the result of nuclear disease was in consonance with experience of nuclear paralysis in general. The evidence of all the relations except the mother was in favor of the deviation having been present before the fall. Probably the lesion was at first an irritative one and caused the nystagmus for the first two months, and then it passed on to be destructive, but destructive only in such a degree as to lame without absolutely paralyzing the left nuclear centre for the third and sixth nerves. This seemed to be the only recorded case of congenital conjugate lateral deviation.

MR. DOYNE had seen an almost exactly similar case in a boy aged 9. It was found on examination by test types that the head, which was at first straight, gradually turned to one side. On close inspection it was seen that shortly after the attention was directed to the object nystagmus began, and then the head turned to the left, the eyes remaining fixed. There had been lagging of one leg in walking for two years, but it was not known if the ocular condition was congenital.

MR. LAWFORD mentioned that there was at least one case on record which seemed to bear upon the case just narrated. The case was that of a man who all his life had conjugate deviation of the eyes to the right. After his death it was found that the left internal rectus was absent, and that the right was exceedingly ill-developed. It was possible that a similar condition existed in Mr. Swanzy's patient.—*British Medical Journal*, Nov. 17, 1888.

---

***Treatment of Tabes Dorsalis.***—In the last few years STEMBO has had 39 cases of tabes under his care, and says (*Berliner klinische Wochenschrift*, Oct. 29, 1888) that the sooner treatment can be begun the better the result to be expected, since some cases are certainly curable. While syphilis may be a predisposing, and a proximate, cause of locomotor ataxia, this fact does not seem to be of value in the treatment of the disease.

Of Stembo's 39 cases, 24 were syphilitic, but none of these were benefited by antisyphilitic treatment. Iodide of potassium did not help any of the cases, nor were good results obtained from arsenic, belladonna, ergot, or strychnine. Antifebrin in full doses was useful in relieving pain—much better than antipyrin and phenacetin. Cocaine gave good results in gastric crises. As to hydrotherapy, Stembo believes that the bathing resorts with warm baths are preferable in the early stages, especially for anæmic and weak patients. In more advanced stages, with strong patients, in whom an increased supply of blood is desired, cold water may be used; but care should be taken that the matter is not overdone. Cold sea-baths must be given with especial caution.

While electricity is decidedly the best means of treating tabes, it must be used with caution, and never by the patients. Stembo uses, in some cases, the constant current applied to the back, followed by the faradic current, or by franklinic electricity applied to the back. In other cases De Watteville's method of using the galvanic and faradic currents simultaneously is employed.

---

***Electricity in Exophthalmic Goitre.***—In the *Therapeutische Monatshefte* for October, 1888, PELZER reports the case of a woman, aged 42, who had suffered from nervous palpitation of the heart for several years. An examination a year before the case came under treatment showed slight exophthalmos and struma, with great palpitation. Under various methods of internal treatment the symptoms grew worse. All internal treatment was then abandoned, and treatment by the constant current begun, one pole being applied over the heart, and the other over the intersterno-cleidomastoid fossa, the séances being of ten minutes' duration. Subsequently the current was passed transversely through the spinal column. During the night an ice-bag was placed over the heart. There was scarcely any noticeable change during the first five weeks, but rapid improvement then set in, first shown by the slowing of the pulse, then by diminution of the exophthalmos, and finally by the retrogression of the struma. After six months' treatment the exophthalmos had entirely disappeared, and the patient's general condition was normal in every respect.

---

***Fractures of the Epicondyles.***—CHARCOT and FRAUCHET record two cases diagnosticated as fractures of the epicondyles of the elbow, and draw the following conclusions:

1. Although Malgaigne denied the existence of such an injury, fracture of the epicondyles does exist. As yet it is only a pathological rarity, but this is because clinicians have not had their attention drawn sufficiently to this complication of fracture of the elbow.

2. The diagnosis of this epiphyseal fracture is most difficult in the first days after the accident, on account of the swelling, the large amount of extravasated blood surrounding the elbow, and the acute pain caused by palpation.

3. The prognosis of the injury is serious. The fracture of the epicondyles helps to make the luxation of the elbow irreducible, and in consequence of the contraction of the adhesions the torn epiphysis may interfere to a great extent with the movements of rotation of the head of the radius.—*Archives de Médicine et de Pharmacie Militaires*, November, 1888.

---

***Salt Infusion in Acute Anœmia.***—DR. C. MOREL, of Lausanne, reports a case of acute anæmia successfully treated by transfusion of a saline solution. The case was that of a woman, aged 39, who in the fifth month of her eleventh pregnancy became ill with gastritis, with high fever. The fever soon caused abortion, followed by profuse uterine hæmorrhage and uncontrollable vomiting. As the patient's condition grew worse rapidly, a litre and a half of a saline solution (a 6 per cent. solution of common salt in distilled water, heated to 37° C.) was injected into the central portion of the median cephalic vein. The injecting apparatus consisted of a simple glass vessel, to which a piece of india-rubber tubing with a metal cannula was attached. The injection caused immediate improvement. The pulse, which had been irregular and almost imperceptible, became stronger and steadier. Dr. Morel hopes that transfusion of saline fluids will supercede blood-transfusion, which is much more dangerous and far less advantageous.

---

***Bichloride of Mercury in Obstetrics.***—Sommer (*Charité Annalen* xiii Jahrgang) reports the results of the use of bichloride of mercury in 5,027 births. The strength of the solution used has been lessened from 1:1000 to 1:4000 for injection. Nineteen cases of mercurial intoxication occurred, with 1 death. Of these cases, 1 resulted from vaginal douches before and after labor; 4 from vaginal douches during the puerperal period; 4 from washing out the uterus after labor; and 10 from repeated uterine douches in the puerperium. It will be observed that intra-uterine douches are most dangerous.

The lowest septic mortality before the use of bichloride was 5 per cent.; during its use from 0.17 to 0.34 per cent.

Sommer believes that 1:5000 is the best solution for injections. For intra-uterine douches 3 to 5 per cent. Carbolic acid solutions should be used. For disinfecting the hands, 1:1000 bichloride solutions.—*Amer. Jour. Med. Sc.*, January, 1889.

---

***Menthol and Iodoform in Surgery.***—At a recent meeting of the Medico-Pharmaceutical Society of Bern Dr. Girard said that he had been led to try a mixture of equal parts of menthol and iodoform, in the form of a dry powder, in 14 cases of scraping out and resection of tuberculous bones and soft parts, the wound being either rubbed or plugged with the powder. In every case the wound healed more rapidly and kindly, and the whole course of the cases seemed to be more favorable than in another series of similar cases in which iodoform was used alone. The menthol causes but trifling pain and local irritation, while it is said to be the best deodoriser of iodoform.

Dr. de Giacomi said that he had used a 20 per cent. solution of menthol in a case of lupus of the ear with satisfactory results.

---

***Myrtol as a Respiratory Disinfectant.***—Eichhorst reports several cases in which he has used myrtol internally as a disinfectant of the respiratory tract. A capsule containing 15 centig. of myrtol will produce a distinct odor of the drug upon the breath within an hour after swallowing it. In the treatment of putrid conditions of the lungs two of these capsules should be given every two hours. The amount of expectoration diminishes, and the general condition improves. So far as could be seen, the drug had no influence on the development of tubercle bacilli.—*Wiener Med. Presse*, No. 42, 1888.

---

***Ergot in Heart-Disease.***—Rosenbach recommends ergot in the treatment of heart disease, to increase the activity of the walls of the bloodvessels, and to increase the pressure in the arterial system. He has found it useful in aortic regurgitation in "idiopathic" dilatation, and in cases of arterio-sclerosis. Ergot regulates the pulse, diminishes dyspnœa and palpitation, but has no effect on the œdema. It may be given in small doses every two or three hours.

---

***Oleum Origanum in Epistaxis.***—Louis Fischer recommends the use of oil of origanum in epistaxis. He gives 5 drops three times a day, an hour after meals, and gradually increases the dose. One patient took 12 drops three times a day. On account of its disagreeable taste and smell it is best given in emulsion or in gelatine capsules.—*Medical Record*, November 17, 1888.

***Helleborin as a Local Anæsthetic.***—VITTOVIO and ELVIDIO, by using aqueous solutions of helleborin instilled under the eyelids in contact with the cornea, have obtained anæsthesia of the cornea that lasted longer than the anæsthesia caused by cocaine. At the same time, they say, the mobility of the eye lid was preserved, there was no dilatation of the pupil, and intraocular tension was not modified. Helleborin also caused local anæsthesia when used subcutaneously, but it produced cardiac disturbance. The drug has a powerful action on the heart, and should be used with great caution.—*Gazette Hebdomadaire*, Sept. 21, 1888.

---

***Antiseptic Pastilles in Diphtheria.***—VAUMOND recommends the following as prophylactic in diphtheria, especially for children that cannot gargle, and in whom pharyngeal applications are difficult:

| | |
|---|---|
| Boracic acid | 20 parts |
| Benzoate of soda | 1 part |
| Oil of thyme | ½ to 1 part |
| Biborate of soda | 20 parts |
| Citric acid | 12½ parts |
| Oil of lemon | 1½ parts |
| Oil of mint | 1½ part M. |

Each pastille should weigh 30 grs., and contain gr. ⅓ boric acid, and gr. $\frac{1}{30}$ each of benzoate of soda and of oil of thyme. Glycerine, water, gum and sugar are used as a basis and solvent, and gelatine is used to give the mass consistence.—*Wiener Med. Presse*, No. 42, 1888.

---

***Butyl-chloral in Trigeminal Neuralgia.***—There are but few drugs that have an action only on the region of a nerve. One of these, says O. LIEBREICH, is butylchloral, which in doses of from 1 to 2 or 3 grams causes, when taken internally, anæsthesia in the course of the trigeminal nerve. Its action in tic douloureux is not lasting, but in rheumatic pains of the face, pains due to trauma, toothache caused by pulpitis or periostitis, butylchloral acts in a satisfactory manner. The taste of the drug is unpleasant, and it is but slightly soluble. The following is a useful formula:

| | |
|---|---|
| Butylchloral | grams 2, 3, or 5 |
| Spts. vini rectif. | " 10 |
| Glycerine | " 20 |
| Aq. destill. | 120 |

Take 3 or 4 dessertspoonfuls.

The quantity to be taken depends on the intensity of the pains and on the patient.—*Internat. Klin. Rundschau*, Dec. 16, 1888.

---

***Treatment of Eczema.***—KAPOSI recommends the following formula as an application for eczema:

| | |
|---|---|
| Naphthol | 5 grams |
| Black soap | 50 grams |
| Powdered chalk | 10 grams |
| Prepared lard | 100 grams |

The parasites are immediately destroyed by this ointment. Different forms of eruptions, especially scabby eczema, are rapidly cured. This ointment should be rubbed in twice daily. It is free from odor, and does not stain linen.

---

***Injections of Ox-Gall in Hydatids.***—JUAN MERCANT reports a case of hydatids of the thigh, from which acephalocysts were discharged by irrigation with a 6 per cent. boric acid solution and injections of ox-gall mixed with an equal quantity of lukewarm water, Three injections were sufficient to expel all the hydatids with their membranous envelopes.—*Medical Chronicle*, Nov., 1888.

---

***Camphor and Ammonium Salts as Stimulants.***—From some experiments BINZ concludes that camphor is a good stimulant to the respiratory function when failure is threatened, as in opium poisoning. Chloride of ammonium is a stimulant to the nervous system, respiration and blood-pressure being increased by a fifth or a quarter in a few minutes.—*Deutsche Med. Wochenschrift*, November 8, 1888.

***What Medicines may be given to Nursing Mothers?***—Fehling has opened an important field of inquiry by a series of experiments to determine what drugs may be safely given to nursing mothers. He found that salicylate of sodium was dangerous to the infant when given to the nurse in doses as large as gr. 45 daily. Iodide of potassium may be given in doses of gr. 3 daily.

Iodoform enters the system of the infant more readily through the nurse than when given to the child. Even when the wounds of the mother were dressed with iodoform, iodine was found in the child's urine.

Fehling found that mercurial salts given to the mother affect the child very slightly if at all.

It was found that 25 drops of tincture of opium (German Pharmac.) and gr. $\frac{1}{10}$ to $\frac{3}{10}$ of morphine could be safely given to the mother.

Chloral may be given in doses of gr. 23 to 45. Atropine affects the child very quickly, even in small doses.

Fehling denies that salads and acids have an injurious effect on the child.

---

***Glycerine in Constipation.***—In a recent number of the *Hospital Gazette* Dr. James D. Staple says he has given glycerine injections more than a hundred times, the quantity injected being ʒj for children, and ʒij for adults. As a rule the bowels acted within fifteen minutes, but in some cases half an hour elapsed, and in two cases the injections had to be repeated. The absence of pain, and the ease with which the enemata may be given, the rapidity of their action, and the absence of any griping, give glycerine enemata a distinct advantage over aperient medicines administered by the mouth.

Glycerine acts equally well, though not so rapidly when given by the mouth, in teaspoonful doses, about every half hour. The effects are particularly good in cases in which the colon is impacted with hardened fæces, the glycerine lubricating the masses so that they are evacuated without pain. Equal quantities of glycerine and castor oil, in teaspoonful doses, also act well.

---

***Treatment of Puerperal Septicœmia.***—Runge (*Archiv für Gynäkologie*, Band 33, Heft 1) has continued to employ his method of treating puerperal septicæmia by the free use of alcoholics, forced feeding, tepid baths, and the omission of antipyretic drugs. His cases amounted to 20, with 15 recoveries: of the 5 fatal cases 4 died from vomiting, which could not be controlled. Runge observed that the use of the bath was followed by a gain in appetite and often by a period of sleep. The alcoholics given were heavy wines and cognac, one prtient averaging half a bottle of port and five ounces of cognac daily for twelve days. Symptoms of intoxication rarely appeared, and were considered favorable signs, as indicating a lessening of the activity of the septic process. Hyperpyrexia was treated by baths and alcoholics daily.—*American Journ. Med. Sc.*, January, 1889.

---

***Morphine in Puerperal Eclampsia.***—Veit, after having used other methods of treating puerperal eclampsia, now relies on morphine, hypodermically, in large doses. The first dose is usually gr. $\frac{3}{5}$, followed by half as much when required. It is generally necessary to give from gr. $1\frac{1}{5}$ to 3 in from 4 to 7 hours—the drug being pushed to the production of narcosis. For the renal complications of eclampsia hot baths are best, followed by packs. Pilocarpine may induce pulmonary œdema. —*Sammlung Klin Vorträge*, No. 304.

---

***A Simple Expectorant Mixture.***

Terpine..............10 grams
Glycerine............60 grams

Dissolve by gentle heat. The dose is regulated by the amount of terpine the physician wishes to give. It may be given in any syrup.

